# A Tale of Two Studies on Auditory Training in Children: A Response to the Claim that ‘Discrimination Training of Phonemic Contrasts Enhances Phonological Processing in Mainstream School Children’ by Moore, Rosenberg and Coleman (2005)

**DOI:** 10.1002/dys.1470

**Published:** 2014-01-27

**Authors:** Lorna F Halliday

**Affiliations:** University College LondonLondon, UK

**Keywords:** auditory training, perceptual learning, phonological awareness, children, randomized controlled trial

## Abstract

In a previous article, Moore, Rosenberg and Coleman (*Brain and Language*, 2005, 94, 72-85) reported evidence for significant improvements in phonological awareness in mainstream children following 6 h of exposure to a commercially available phoneme discrimination training programme, but not in a control group. In a follow-up study, we failed to replicate this finding, despite using an almost identical training programme (Halliday, Taylor, Millward, & Moore, *Journal of Speech, Language, and Hearing Research*, 2012, 55, 168-181). This paper directly compares the methods and the results of the two studies, in an effort to explain the discrepant findings. It reports that the trained group in Moore *et al*. (2005) showed significantly greater improvements in phonological awareness following training than the trained group in Halliday *et al*. (2012). However, the control group in Halliday *et al*. (2012) showed significantly greater improvements in phonological awareness than the control group in Moore *et al*. (2005). The paper concludes that differences in the randomization, blinding, experimenter familiarity and treatment of trained and control groups contributed to the different outcomes of the two studies. The results indicate that a plethora of factors can contribute to training effects and highlight the importance of well-designed randomized controlled trials in assessing the efficacy of a given intervention. © 2014 The Authors. *Dyslexia* published by John Wiley & Sons, Ltd.

## INTRODUCTION

With the advent of learning technologies, the fledgling field of ‘educational neuroscience’ is fast becoming a cornerstone of educational policy and practice. Educators are encouraged to follow an evidence-based approach, seeking out intervention studies with a view to informing practice. However, with an explosion of research papers published in the last decade, the task of interpreting that research is no mean feat (Howard-Jones, 2009). The difficulty in ascertaining the efficacy of a given intervention stems from the fact that there are multiple factors that can influence outcome. These include maturational, placebo and test–retest effects, as well as regression to the mean, and differences in participant characteristics and drop-out rates both within and between different studies. This has led to the call from several researchers for the introduction of the randomized controlled trial (RCT) as the ‘gold standard’ test of neuroscientific interventions (e.g. Snowling & Hulme, 2011).

The RCT confers significant advantages over and above other intervention designs (Troia, 1999). In randomly assigning participants to different conditions, it minimizes the risk of pre-existing differences between groups and of differences in the treatment of those groups outside the intervention. A no-intervention (NI) group should be used, to assess maturational changes that might occur during the intervention, as well as test–retest/practice effects. In addition, it is preferable to also include one or more control intervention groups, who receive broadly similar amounts of attention and spend similar amounts of time on task. This allows researchers to assess whether a particular intervention is more effective than simply giving a child more attention (e.g. the ‘Hawthorne effect’) or raising their expectations (i.e. ‘placebo effects’). Finally, in order to minimize expectancy effects, it is preferable for both the experimenter and the child to be blind to the intervention received. Currently, very few neuroscientific intervention studies adopt such a strict set of criteria. However, studies that fail to do so can be associated with exaggerated treatment effects (see Altman *et al*., 2001). Consequently, educators are often left with the challenge of trying to disentangle changes in behaviour arising from a given intervention from those arising from methodological inadequacies in a study's design.

A case in point comes from the literature on auditory training in children. This literature originates from an influential theory that states that developmental disorders such as dyslexia and specific language impairment (SLI) arise from a low-level deficit in processing auditory information (e.g. Tallal, 2004). According to this theory, these deficits lead to the development of poorly specified phonological representations that, in turn, lead to difficulties in learning to read in the case of dyslexia or in acquiring the rules of oral language, in the case of SLI. There is now a considerable amount of evidence to suggest that many children and adults with dyslexia and/or SLI have deficits in auditory processing, although the causal nature of this relationship is yet to be determined (for review, see Rosen, 2003). Nevertheless, these findings have led to the development of a number of commercially available computer-based programmes (e.g. Fast ForWord Language®, FFW-L Scientific Learning Corporation, 1998; Earobics, Houghton Mifflin Harcourt, 2000; Phonomena^©^, MindWeavers Ltd., 2002), designed to treat disorders such as SLI and dyslexia and to improve speech and language skills in typically developing children (e.g. Diehl, 1999; Hayes, Warrier, Nicol, Zecker, & Kraus, 2003; Moore, Rosenberg, & Coleman, 2005; Morrison, 1998; Pokorni, Worthington, & Jamison, 2004).

Although now widely used as tools for improving oral and written language in children, an outstanding issue is whether or not such auditory training programmes actually work. Here, the case for FFW-L has become increasingly controversial. To date, there are now three RCTs that have included FFW-L as one of the interventions for children with the following: (1) SLI (Gillam *et al*., 2008); (2) language and reading impairment (Pokorni *et al*., 2004); and (3) mixed receptive and expressive SLI (Cohen *et al*., 2005). However, none of these studies has reported a significant remedial advantage of FFW-L, in terms of performance on standardized measures of receptive and expressive language, relative to the following: (1) other computer-based interventions designed to improve language but without modified speech (e.g. Earobics; Cohen *et al*., 2005; Gillam *et al*., 2008; Pokorni *et al*., 2004); (2) other computer-based interventions not designed to improve language (Gillam *et al*., 2008); (3) therapist-centred language interventions (Gillam *et al*., 2008); or (4) ‘waiting list’ controls who received their regular speech and language intervention services during the training period (Cohen *et al*., 2005). Moreover, although all groups of children in the studies of Cohen *et al*. (2005) and Gillam *et al*. (2008) made significant gains in language with (Gillam *et al*., 2008), and without additional gains in auditory processing (Cohen *et al*., 2005), those in the study of Pokorni *et al*. (2004) did not. Together, these findings question the efficacy of FFW-L auditory training at improving language abilities in children with language impairment, certainly over and above that of other (auditory and nonauditory; language and nonlanguage) interventions and, perhaps, over and above that of doing no training at all.

These negative findings have inevitably led to controversies in the literature. One of the difficulties that educators face in interpreting conflicting findings is that it is not always clear *why* a particular study has found a particular result. Studies tend to use quite different methods, different training paradigms and different outcome measures to assess particular interventions. This paper directly compares the outcomes of two intervention studies on auditory training in children, which were very similar in method and design, but which differed in the extent to which they adopted the RCT criteria (see Altman *et al*., 2001). The case study is used to illustrate how relatively minor differences in the method and design of a given study can lead to very different interpretations regarding the efficacy of an intervention.

### The Case Study

In 2005, Brain and Language published a study by Moore *et al*., which was designed to test the efficacy of a commercially available phoneme discrimination (PD) training programme (‘Phonomena’^©^, Oxford, UK., MindWeavers, Ltd., 2002). Phonomena was a computer-based auditory training programme designed to train the discrimination of English phonemes in order to accelerate language learning in multiple target groups (typically developing children and children with language and other learning difficulties at any age who were learning English as their first language, as well as children and adults learning English as a second language). The parent company, MindWeavers Ltd, has since ceased trading. In Moore *et al*. (2005), 18 typically developing 8- to 10-year-old children were recruited from a single tutor group of a mainstream primary school in Oxfordshire, UK. These children were given three 30-min sessions of training per week for 4 weeks on a psychophysical PD task. Children were trained on 11 different phoneme contrasts spoken by a single male speaker, and task difficulty (the similarity between the two phoneme contrasts) was adapted in response to task success. A second group of children was recruited from the parallel tutor group in the same school (*n* = 12) but did not receive any training (‘NI’ control group). Immediately before (pre-training) and after (post-training) training, both groups were given a battery of standardized tests of phonological awareness (the ‘Phonological Assessment Battery’ (PhAB); Frederickson, Frith, & Reason, 1997) and a word discrimination task (the ‘Word Discrimination Test’; Rosenberg & Moore, 2003), and the pre-training to post-training improvement in performance was compared between groups. The trained group was also retested 5–6 weeks after training (delayed test). During training, Moore *et al*. (2005) found that the trained group showed little in the way of improvements on the PD tasks upon which they were trained. However, this same group showed significant improvements between pre-training and post-training on the four subtests of the PhAB (Alliteration, Rhyme, Spoonerisms, and Nonword Reading) and the Word Discrimination Test, and these were maintained 5–6 weeks later. In contrast, the control group did not show improvements on any of the language assessments between pre-training and post-training.

In a recent study, we used a similar training programme to Moore *et al*. (2005) to examine auditory learning in children (Halliday, Taylor, Millward, & Moore, 2012). In this study, we recruited 86, 8- to 10-year-old typically developing children from the form groups of two primary schools in Nottinghamshire, UK. These children were quasirandomly assigned to one of four groups. One group was trained on a PD discrimination task using stimuli that were identical to those used in the Moore *et al*. (2005) study (the ‘PD group’; *n* = 22). A second group was trained on an auditory frequency discrimination task designed to match the PD task in terms of task demands (‘AFD group’; *n* = 22), and a third group was trained on a visual analogue of the AFD task, a visual frequency discrimination task (‘VFD group’, *n* = 20). Finally, a fourth group of children received no intervention and participated in normal school activities during training (‘NI group’, *n* = 22). As in the Moore *et al*. (2005) study, the three trained groups were trained on 11 different stimulus contrasts, for three 30-min sessions per week for 4 weeks. Immediately before and after training, all children were tested on three of the subtests from the PhAB (Alliteration, Rhyme and Spoonerisms), a test of Nonword Repetition from the A Developmental NEuroPSYchological Assessment (Korkman, Kirk, & Kemp, 1998), and two tests of Word and Nonword Reading from the Test of Word Reading Efficiency (Torgesen, Wagner, & Rashotte, 1999). During training, we found that both the PD group and the AFD group showed significant learning on the tasks upon which they were trained. However, unlike Moore *et al*. (2005), we found that none of our four groups differed significantly from each other at post-training on any of the language measures, after controlling for pre-training scores. Crucially, this comparison included the PD group and NI group that were comparable to those used in the Moore *et al*. (2005) study.

Because of the similarities between the two studies, an outstanding question is why we did not replicate the results of Moore *et al*. (2005). Here, we directly compare the methods and results of Moore *et al*. (2005) and Halliday *et al*. (2012) to assess whether the discrepant results can be attributed to differences in the participant characteristics, training programme and/or design of the two studies.

## METHODS

Detailed methods of the two training studies can be found in Moore *et al*. (2005) and Halliday *et al*. (2012). Key differences in the training methods and designs of the two studies are summarized in Tables 1 and 2, respectively.

**Table 1:** Comparison of training programmes for Moore *et al*. (2005) and Halliday *et al*. (2012)

Training programme		Moore *et al*. (2005)	Halliday *et al*. (2012)
Training game	Game format	‘Sound Game’ from ‘Phonomena’ (MindWeavers plc.). Listeners interact with a cartoon dinosaur tutor character and two cartoon furry cavemen student characters in a classroom setting	‘System for Testing Auditory Responses’, (MRC Institute of Hearing Research). Listeners interact with one of five different animal or human cartoon characters, on one of five different backgrounds
	Sound sets	*n* = 11 from ‘Phonomena’ spoken by a single male speaker of south-east British English	As Moore *et al*. (2005)
	Response paradigm	3-interval, 2-alternative forced choice XAB	3-interval 3-alternative forced choice oddball
	Adaptive procedure	3 down, 1 up adaptive staircase	1 down, 1 up, followed by 3 down, 1 up adaptive staircase
	Stimulus presentation	Stimuli moved inwards and outwards from two endpoints of the continuum (variable standard presentation)	One stimulus remained fixed, whilst the other moved downwards and upwards along the continuum (fixed standard presentation)
	No. trials per game	60	25
	Reward procedure	Non-training game, ‘3's Company’ from ‘Phonomena’, where players interactively use a catapult to throw cartoon faces at a wall. Experimenter encouragement. Stickers and prize reward scheme	Choice of character and background for launch of next successive game. Collection of tokens as indication of past performance success. Experimenter encouragement. Stickers, certificates, and prize reward scheme
Amount of training	Mean ± standard deviation no. trials	1656.67 ± 313.56	1400.00 ± 234.52
	Range of no. trials	1140–2460	925–1900

**Table 2:** Comparison of design of Moore *et al*. (2005) and Halliday *et al*. (2012)

Design	Moore *et al*. (2005)	Halliday *et al*. (2012)
Group assignment	Deterministic. Participants assigned to groups based on tutor group membership	Quasi-, stratified randomization. Participants assigned to groups on a quasirandomized basis (children with visual anomalies not assigned to the visual frequency discrimination group), stratified according to gender, year group (year 4 versus year 5), and native-English status
Existence of a control group	No-intervention control group only	No-intervention control group; auditory training control group; visual training (placebo) control group
‘Blindness’ of (a) participants; (b) experimenters to group membership	Neither (a) participants nor (b) experimenters blind to group membership	(a) Participants in three training groups (phoneme discrimination group; auditory frequency discrimination group; visual frequency discrimination group) blind to training group membership. Participants in the no-intervention group not blind no-intervention status. (b) Experimenters not blind to group membership of participants
Blindness of experimenters to pretraining scores at posttraining	Experimenters not blind to pretraining scores at posttraining	Experimenters blind to pretraining scores at posttraining
Similarity of treatment of trained and no-intervention groups	Phoneme discrimination group, but not no-intervention group, rewarded with prizes	Both phoneme discrimination and no-intervention groups rewarded with prizes
Experimenter familiarity	Phoneme discrimination group familiarized with experimenters at pre- and post-training and during training. No-intervention group familiarized with experimenters at pre- and post-training only	Phoneme discrimination group familiarized with experimenters at pre- and post-training and during training. No-intervention group familiarized with experimenters at pre- and post-training, and during training for ∼5 min per week on a group basis and 9 times per week during participant collection

In order to facilitate direct comparison between studies, we focused solely on the two PD groups (‘PD-Moore’ and ‘PD-Halliday’) and the two NI groups (‘NI-Moore’ and ‘NI-Halliday’) from Moore *et al*. (2005) and Halliday *et al*. (2012). Pre-training to post-training improvement in phonological awareness skills was assessed using the three cognitive tests that overlapped between the two studies. These were the Alliteration, Rhyme and Spoonerisms subtests from the PhAB (Frederickson *et al*., 1997). As no follow-up data were available for Halliday *et al*. (2012) (i.e. a session comparable with the ‘delayed’ session in Moore *et al*., 2005), only pre-training to post-training change will be considered here.

Kolmogorov–Smirnov tests showed that ceiling effects meant that the pre-training and post-training scores for the Alliteration subtest were nonnormally distributed for all groups bar one (pre-training Alliteration for the NI-Moore group). Consequently, for the Alliteration subtest only, data were analysed using nonparametric statistics.

## RESULTS

### Participant Characteristics

Participant characteristics for the two studies are shown in Table 3. Chi-squared analyses showed that neither the two PD groups (PD-Moore; PD-Halliday) nor the two NI groups (NI-Moore; NI-Halliday) differed significantly in gender or the proportion of nonnative English speakers. Groups PD-Halliday and NI-Halliday were significantly older than groups PD-Moore and NI-Moore, respectively. The PD-Moore and PD-Halliday groups did not differ significantly in their pre-training scores on the Alliteration, Rhyme or Spoonerisms subtests. The NI-Moore and NI-Halliday groups did not differ significantly at pre-training on Alliteration or Spoonerisms. However, the NI-Halliday group had significantly higher pre-training scores on the Rhyme subtest than the NI-Moore group.

**Table 3:** Comparison of participant characteristics for Moore *et al*. (2005) and Halliday *et al*. (2012)

Participant characteristic	Group	Study		Statistic	*p*-value
		Moore *et al*. (2005)	Halliday *et al*. (2012)		
Participant *n*	PD	18	22		
	NI	12	22		
Ratio of males:females	PD	7 : 11	12 : 10	*χ*^2^ (1) = 0.97	0.324
	NI	6 : 6	10 : 12	*χ*^2^ (1) = 0.06	0.800
Age (years) at pretraining (M ± SD)	PD	9.09 ± 0.32	9.62 ± 0.62	*t*(38) = −3.26	0.002
	NI	9.08 ± 0.33	9.49 ± 0.59	*t*(32) = −2.24	0.032
Nonnative English	PD	1	4	*χ*^2^ (1) = 1.44	0.230
	NI	3	2	*χ*^2^ (1) = 1.57	0.211
Alliteration at pretraining (M ± SD)	PD	8.72 ± 2.14	8.75 ± 1.29	U = 192	0.859
	NI	8.64 ± 2.52	9.05 ± 1.21	U = 111	0.466
Rhyme at pretraining (M ± SD)	PD	13.5 ± 5.63	15.55 ± 5.23	*t*(38) = −1.19	0.242
	NI	11.58 ± 5.12	16.73 ± 4.97	*t*(32) = −2.85	0.008
Spoonerisms at pretraining (M ± SD)	PD	12.50 ± 7.32	16.36 ± 7.77	*t*(38) = −1.61	0.117
	NI	13.75 ± 6.54	16.27 ± 7.92	*t*(32) = −.094	0.354

NI, no-intervention; PD, phoneme discrimination; M ± SD, mean ± standard deviation.

Results of the cognitive tests are reported as raw scores.

### Training

**Figure 1:**
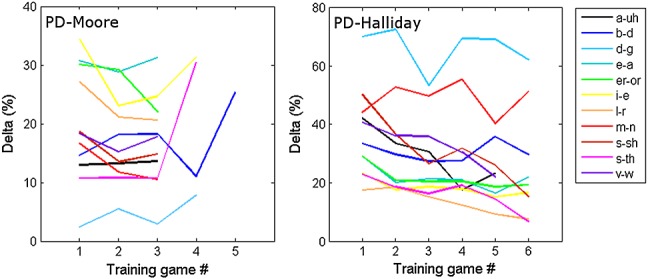
Mean thresholds during training for each of the 11 stimulus sets as a function of sequential training game number, for the PD-Moore group and PD-Halliday group. Here, we include data only for those stimulus sets and training game blocks that were completed by at least 25% of the members of each group. Reconstructed with permission from Moore *et al*. (2005) and Halliday *et al*. (2012).

Differences in the psychophysical procedures meant that it was not possible to quantitatively compare the amount of learning between the two studies (Table 1). Differences between the studies were therefore assessed first qualitatively, by inspecting the slopes of the training curves on the PD task between the two studies, and second quantitatively, by comparing the amount of training received by the two groups. Figure 1 shows the change in thresholds on the PD task as a function of training game number for the PD-Moore and PD-Halliday groups. It is evident from Figure 1 that these two groups showed marked differences in their online training. Most notably, children trained in the Moore *et al*. (2005) study showed very little evidence for learning on the PD task, as is reported in their paper. For some of the stimuli, this was because children could discriminate them easily from the first game (i_e, e_a, er_or and l_r). However, for the others, performance remained modest throughout, apart from the stimulus set d_g that ‘proved extremely difficult for children to discriminate’ throughout (Moore *et al*., 2005, p. 79). Children in the study of Halliday *et al*. (2012) in contrast showed significant learning on the PD task, although this was confined to the stimulus sets ‘a_uh’, ‘e_a’, ‘l_r’, ‘s_sh’ and ‘s_th’ (see Halliday *et al*., 2012, p 175).

Differences in the amount of training received by the two trained groups are also shown in Table 1. Despite both groups receiving 6 h of training in total and this being intermixed with nontraining games in the Moore *et al*. (2005) study (Table 1), the PD-Moore group completed significantly more PD training trials than the PD-Halliday group, *t*(38) = −2.96, *p* = 0.005.

### Pre-training to Post-training Improvement

For ease of comparison between the two datasets, the age-equivalent scores reported in the study of Moore *et al*. (2005) were converted to raw scores. Analysis of raw, rather than age-equivalent scores is appropriate here as raw scores maintain a lot of information that is thrown away by age-equivalent scores (i.e. as occurs when children with different raw scores are assigned the same age-equivalent score; see Snowling & Hulme, 2003, for further elaboration on this point).^1^ As outlined above, there were slight differences in the scores of participants at pre-training between the two studies. Consequently, in order to control for these potential differences, Mann–Whitney tests were used to assess between-group differences in pre-training to post-training changes in raw scores for the Alliteration subtest. For the Rhyme and Spoonerisms subtests, a series of ANCOVAs were used to assess the differences in raw scores between groups at post-training, controlling for raw scores at pre-training.

**Figure 2:**
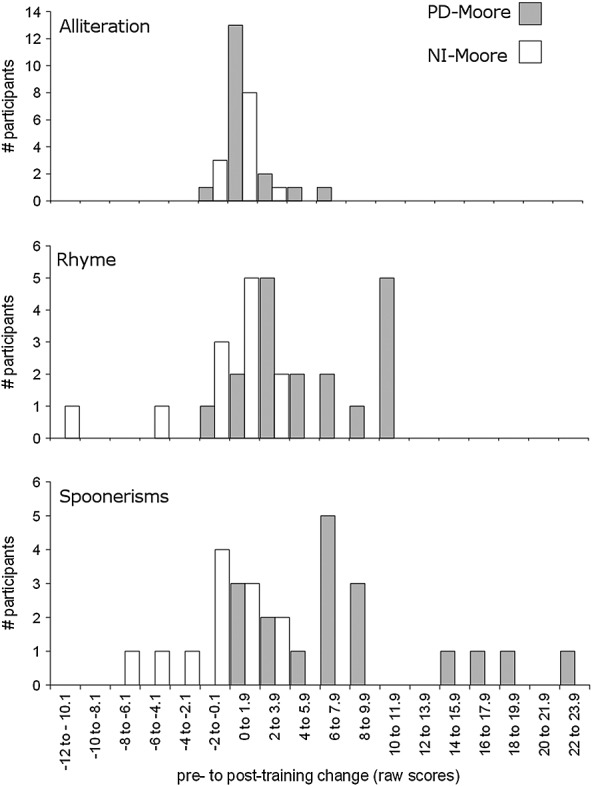
Histograms of pretraining to posttraining change in raw scores on the Alliteration, Rhyme and Spoonerisms subtests of the Phonological Assessment Battery for the phoneme discrimination (PD-Moore) and no-intervention (NI-Moore) groups from Moore *et al*. (2005). A positive score on the *x*-axis indicates pretraining to posttraining improvement.

Figure 2 shows the distribution of pre-training to post-training change in raw scores (post-training score minus pre-training score) for individuals in the two groups in the Moore *et al*. (2005) study, for the Alliteration, Rhyme and Spoonerisms subtests, respectively. A Mann–Whitney test found that the PD-Moore group did not show a greater amount of improvement on the Alliteration subtest between pre-training and post-training than the NI-Moore group, *U* = 80.5, *p* = 0.249. However, the PD-Moore group showed significantly higher post-training scores than the NI-Moore group on both the Rhyme and Spoonerisms subtests, after controlling for pre-training scores, *F*(1, 27) = 76.05, *p* < 0.001 (partial *ŋ*^2^ = 0.74), and *F*(1, 27) = 23.28, *p* < 0.001 (partial *ŋ*^2^ = 0.46), respectively.

**Figure 3:**
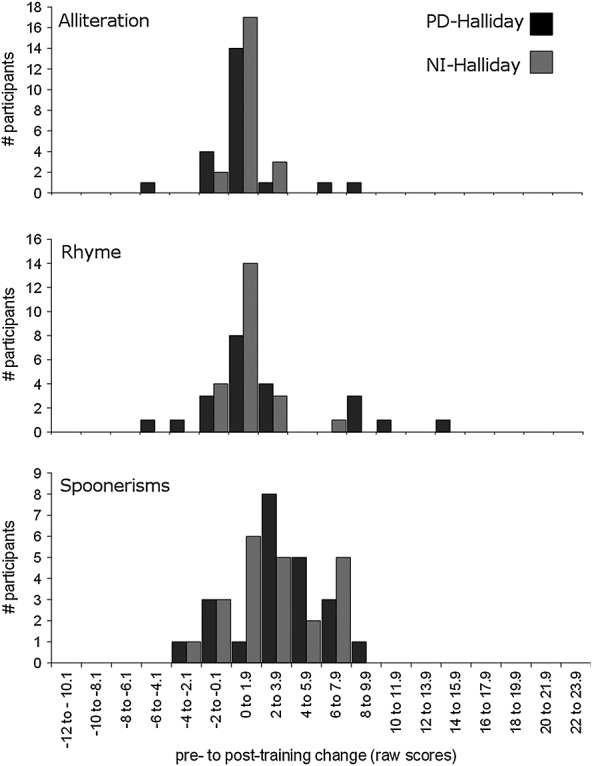
Histograms of pretraining to posttraining change in raw scores on the Alliteration, Rhyme and Spoonerisms subtests of the Phonological Assessment Battery for the phoneme discrimination (PD-Halliday) and no-intervention (NI-Halliday) groups from Halliday *et al*. (2012). A positive score on the *x*-axis indicates pretraining to posttraining improvement.

Figure 3 shows the same data for the study of Halliday *et al*. (2012). The PD-Halliday group did not show significantly more improvement than the NI-Halliday group between pre-training to post-training for the Alliteration subtest, *U* = 215.0, *p* = 0.501. Moreover, there were no significant group differences in post-training scores on the Rhyme, *F*(1, 41) = 0.93, *p* = 0.341 (partial *ŋ*^2^ = 0.02), or Spoonerisms subtests, *F*(1, 41) = 0.05, *p* = 0.829 (partial *ŋ*^2^ = .001) after controlling for pre-training scores.

**Figure 4:**
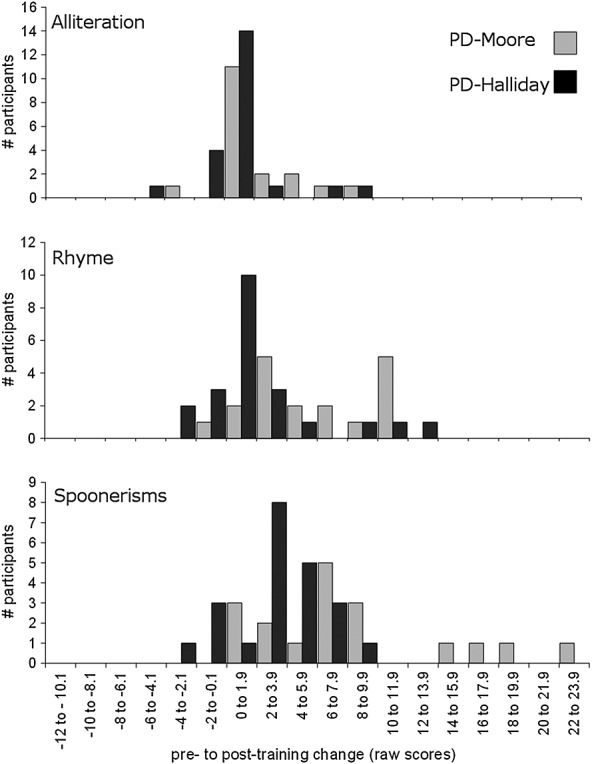
Histograms of pretraining to posttraining change in raw scores on the Alliteration, Rhyme and Spoonerisms subtests of the Phonological Assessment Battery for the phoneme discrimination groups from Moore *et al*. (2005) (PD-Moore) and Halliday *et al*. (2012) (PD-Halliday). A positive score on the *x*-axis indicates pretraining to posttraining improvement.

Pre-training to post-training change following training for the PD groups from the studies of Moore *et al*. (2005) and Halliday *et al*. (2012) is compared in Figure 4. The PD-Moore and PD-Halliday groups did not differ significantly in their amount of improvement on the Alliteration subtest, *U* = 161.0, *p* = 0.325. However, the PD-Moore group showed significantly higher post-training scores than the PD-Halliday group on both the Rhyme, *F*(1, 37) = 6.62, *p* = 0.014 (partial *ŋ*^2^ = 0.15), and the Spoonerisms subtests, *F*(1, 37) = 6.83, *p* = 0.013 (partial *ŋ*^2^ = 0.16), after controlling for pre-training scores.

**Figure 5:**
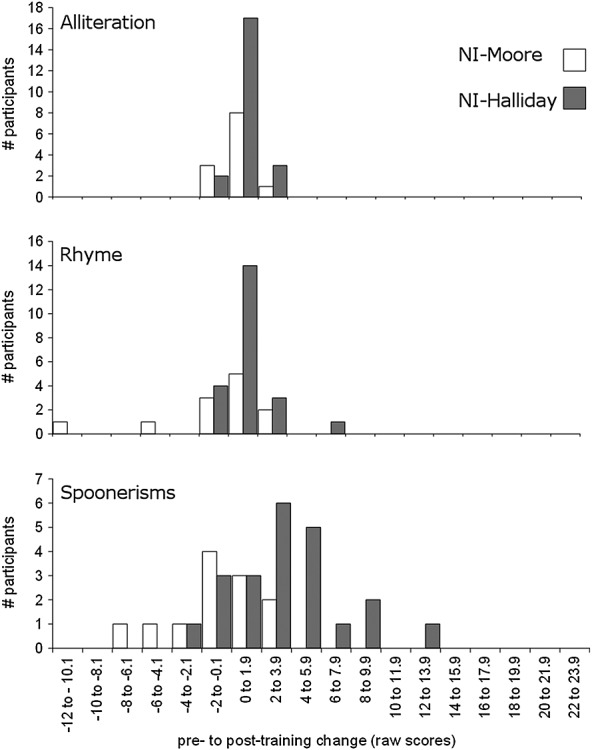
Histograms of pretraining to posttraining change in raw scores on the Alliteration, Rhyme and Spoonerisms subtests of the Phonological Assessment Battery for the no-intervention groups from Moore *et al*. (2005) (NI-Moore) and Halliday *et al*. (2012) (NI-Halliday). A positive score on the x axis indicates pretraining to posttraining improvement.

The change in pre-training to post-training scores is compared in Figure 5 for the two NI groups from the studies of Moore *et al*. (2005) and Halliday *et al*. (2012). The NI-Moore and NI-Halliday groups did not differ significantly in their pre-training to post-training improvement on the Alliteration subtest, *U* = 110.5, *p* = 0.444. However, the NI-Halliday group showed significantly higher post-training scores than the NI-Moore group on both the Rhyme, *F*(1, 31) = 13.85, *p* = 0.001 (partial *ŋ*^2^ = 0.31), and Spoonerisms subtests, *F*(1, 31) = 17.07, *p* < 0.001 (partial *ŋ*^2^ = 0.36), after controlling for pre-training scores.

Finally, given that the groups differed in their proportions of nonnative English speakers (albeit, nonsignificantly), the analyses were repeated after excluding these children. The results remained the same throughout (see Supplemental Information).

## DISCUSSION

The analyses reported in the previous texts directly compared the methods and results of the studies of Moore *et al*. (2005) and Halliday *et al*. (2012) in an effort to explain their discrepant findings. Consistent with the original papers, reanalysis of the data found that the PD group in the study of Moore *et al*. (2005) showed significantly greater improvements in Rhyme and Spoonerisms following training compared with NI controls. However, the PD group in the study of Halliday *et al*. (2012) did not. New analyses showed the following: (1) the PD-Moore group showed significantly greater improvements in Rhyme and Spoonerisms following training than the PD-Halliday group, whereas (2) the NI-Halliday group showed significantly greater pre-training to post-training improvements on these measures than the NI-Moore group. It is possible that these differences may have arisen because of differences in the following: (1) participant characteristics; (2) training programmes; or (3) design of the two studies.

A comparison of the participant characteristics of the two studies indicates that it is unlikely that these differences contributed to the discrepant findings. First, the proportions of nonnative English speakers in each of the two studies did not differ significantly, but even when these children were excluded from the analyses, the results remained the same. Second, children in the study of Halliday *et al*. (2012) were slightly older than children in the study of Moore *et al*. (2005). However, pre-training scores for the two studies did not in general differ significantly. Second, where they did (children in the NI-Halliday group had higher pre-training Rhyme scores than children in the NI-Moore group), this did not lead to diminished pre-training to post-training gain (the NI-Halliday group actually showed a greater degree of pre-training to post-training improvement than the NI-Moore group). Finally, there were greater numbers of children in the study of Halliday *et al*. (2012) compared with the study of Moore *et al*. (2005), particularly in the NI group, possibly leading to an increase in power to detect pre-training to post-training changes in performance. However, an inspection of Figure 5 suggests that this too cannot fully account for the results; here, the distributions of the two groups are clearly different, in that very few of the NI-Moore group showed any improvement at all on either the Rhyme or the Spoonerisms subtests.

Differences in the training methods of the two studies also cannot entirely account for the results. Although the two studies used different training procedures, the 3-interval, 2-alternative forced-choice AXB paradigm used by Moore *et al*. (2005) has been shown to yield comparable thresholds and learning to the 3-interval 3-alternative forced-choice oddball design used by Halliday *et al*. (2012) (Amitay, Irwin, Hawkey, Cowan, & Moore, 2006). Nevertheless, the fact that the latter paradigm requires listeners to hold up to two items in memory (as opposed to one for the AXB task) may have contributed to the greater learning that was observed by Halliday *et al*. (2012) for their PD group. It cannot, however, explain the lesser generalization of this group to the tests of phonological awareness. It is possible that differences in the number of trials per game (25 vs 60) and stimulus presentation paradigms (fixed vs variable) contributed in part to the study outcomes. The longer duration of the training games in the study of Moore *et al*. (2005) may have meant that children spent more time around threshold, that is, listening to difficult discriminations, something that we know is likely to induce learning (Amitay, Irwin, & Moore, 2006). However, this does not explain why the PD-Moore group seemingly showed *less* learning on the PD task. Nevertheless, there is some suggestion that a variable presentation paradigm can yield greater generalization of learning than a fixed presentation paradigm, at least in some listeners (Amitay, Hawkey, & Moore, 2005). In addition, the lack of a standard in the study of Moore *et al*. (2005) will have meant that children in the PD group would have been required to make relative judgements about each stimulus set rather than being able to form and rely upon a memory representation of the standard (i.e. a perceptual anchor). However, none of these differences can explain the greater degree of learning seen in the NI-Halliday group versus the NI-Moore group. Finally, it is possible that the greater degree of training completed by the PD-Moore group contributed to their greater pre-training to post-training improvement in phonological awareness. However, again, the results for the NI groups need explaining.

A more parsimonious explanation is that differences in the designs of the two studies were largely responsible for our failure to replicate the results of Moore *et al*. (2005). There were six differences in the designs of the studies of Moore *et al*. (2005) and Halliday *et al*. (2012). First, whereas children in the study of Moore *et al*. (2005) were assigned to their groups on the basis of tutor group membership, group assignment was pseudorandom in Halliday *et al*. (2012). Second, whereas the study of Moore *et al*. (2005) had a NI control group only, the study of Halliday *et al*. (2012) also included an auditory and visual treatment control group. Third, whereas the children in the training groups of Halliday *et al*. (2012) were blind to expectations about the relative benefits of each training programme, children in the study of Moore *et al*. (2005) were not. Fourth, whereas the experimenters in Halliday *et al*. (2012) were blind to children's pre-training scores at post-training, those in Moore *et al*. (2005) were not. Fifth, whereas only the trained (PD-Moore) group received rewards for participation in the study of Moore *et al*. (2005), all groups received rewards in Halliday *et al*. (2012). Finally, sixth, whereas the NI group in the study of Moore *et al*. (2005) were only exposed to the experimenters at pre-training and post-training, the NI group in the study of Halliday *et al*. (2012) had regular contact with the experimenters during the training period in addition to pre-training and post-training.

How can these design features account for the differences in the findings between these two studies? First, it is possible that the results of the study of Moore *et al*. (2005) were subject to pre-existing differences between the trained and non-trained groups or to differences in the treatment of these two groups between pre-training and post-training. Because group assignment was carried out per tutor group in Moore *et al*. (2005), it was not possible to control for either of these factors. Second, the results may have been due to placebo effects (i.e. improvements in performance owing to participants' knowledge of trained group membership). Because only one trained group was used in Moore *et al*. (2005), children in the PD-Moore group knew they were in the intervention group, and conversely, those in the NI-Moore group knew they were not. In contrast, because more than one training group was included in Halliday *et al*. (2012), children in the three trained groups were unlikely to have had expectations about which intervention was likely to yield performance change. This may explain why children in the PD-Moore group showed significantly greater improvements between pre-training and post-training than children in the PD-Halliday group.^2^ Third, it is possible that the results of the study of Moore *et al*. (2005) were subject to Hawthorne effects (i.e. improvements in performance owing to factors other than the training itself). Children in the PD-Moore group were given more attention and rewards than children in the NI-Moore group and were more familiar with the experimenters at post-training, whereas the groups were treated more similarly in Halliday *et al*. (2012). This would explain why children in the NI-Halliday group showed significantly greater pre-training to post-training improvements than the NI-Moore group. Finally, it is possible that the discrepant findings were attributable to experimenter effects. Although experimenters in Halliday *et al*. (2012) were not blind to children's training group status, they were blind to their pre-training scores, meaning that they were unable to use these as a ‘rule of thumb’ in guiding post-training scores. That Moore *et al*. (2005) did not use such measures suggests that their results may have been more susceptible to experimenter expectancy than those of Halliday *et al*. (2012). This would explain why the PD-Moore group and the NI-Moore groups showed, respectively, significantly greater and lesser pre-training to post-training improvements in phonological awareness than the PD-Halliday and NI-Halliday groups.

The implications for these findings are twofold. First, the results of Halliday *et al*. (2012) suggest that when these design factors are controlled for, 8- to 10-year-old typically developing children trained for 6 h on a PD task with 11 stimulus sets do not show significantly greater improvements in phonological awareness compared with a non-trained control group. However, the results also have wider implications for educational intervention studies in general and the subsequent interpretation of these. They illustrate the importance of random assignment of participants to interventions, attempts to blind both the participants and the experimenters and, in particular, the role of intervention and NI control groups in interpreting the efficacy of a given treatment. Importantly, the findings also emphasize the need for researchers to provide and for readers to examine the raw pre-intervention to post-intervention data. Where a control group either shows little or no overlap in pre-intervention to post-intervention change compared with an intervention group or a detriment in performance over that time, alarm bells should start ringing.

Finally, it is important to note that the findings reported here in no way challenge the efficacy of the Phonomena programme in accelerating language learning in children. Halliday *et al*. (2012) used only a subset of the stimuli included in Phonomena (which includes multiple speakers of the phonemic continua) and used a different training programme to deliver these stimuli. However, like Halliday *et al*. (2012), Moore *et al*. (2005) also used only a subset of the stimuli included in Phonomena. Therefore, arguably, the results of neither study can demonstrate or refute the efficacy of Phonomena. Only the results of a RCT using the Phonomena programme could achieve this.

## NOTES

1 Nevertheless, at the request of one anonymous reviewer, the analyses reported here were repeated using standard rather than raw scores. The results remained the same throughout (see Supplemental Information).

2 Although note that this might conversely predict that all three training groups in Halliday *et al*. (2012) should show placebo effects in the form of greater pretraining to posttraining improvements in performance on the PhAB relative to the NI group. However, this was not the case (see Halliday *et al*., 2012, p. 177).

## Supporting Information

Supporting info item

## References

[b1] Altman DG, Schulz KF, Moher D, Egger M, Davidoff F, Elbourne D, Lang T (2001). The revised CONSORT statement for reporting randomized trials: Explanation and elaboration. Annals of Internal Medicine.

[b2] Amitay S, Irwin A, Hawkey DJC, Cowan JA, Moore DR (2006). A comparison of adaptive procedures for rapid and reliable threshold assessment and training in naïve listeners. Journal of the Acoustical Society of America.

[b3] Amitay S, Irwin A, Moore DR (2006). Discrimination learning induced by training with identical stimuli. Nature Neuroscience.

[b4] Amitay S, Hawkey DJC, Moore DR (2005). Auditory frequency discrimination learning in affected by stimulus variability. Perception & Psychophysics.

[b5] Cohen W, Hodson A, O'Hare A, Boyle J, Durrani T, McCartney E, Watson J (2005). Effects of computer-based intervention through acoustically modified speech (Fast ForWord) in severe mixed receptive-expressive language impairment: Outcomes from a randomized controlled trial. Journal of Speech, Language, and Hearing Research.

[b6] Diehl S (1999). Listen and learn? A software review of Earobics. Language Speech Hearing Services in the Schools.

[b7] Frederickson N, Frith U, Reason R (1997). The phonological assessment battery.

[b8] Gillam RB, Loeb DF, Hoffman LM, Bohman T, Champlin CA, Thibodeau L, Friel-Patti S (2008). The efficacy of Fast ForWord Language intervention in school-age children with language impairment: A randomized controlled trial. Journal of Speech, Language, and Hearing Research.

[b9] Halliday LF, Taylor JL, Millward KE, Moore DR (2012). Lack of generalization of auditory learning in typically developing children. Journal of Speech, Language, and Hearing Research.

[b10] Hayes EA, Warrier CM, Nicol T, Zecker SG, Kraus N (2003). Neural plasticity following auditory training in children with learning problems. Clinical Neurophysiology.

[b11] Howard-Jones PA (2009). Scepticism is not enough. Cortex.

[b26] Houghton Mifflin Harcourt (2000). Earobics.

[b12] Korkman M, Kirk U, Kemp S (1998). A developmental neuropsychological assessment.

[b13] Moore DR, Rosenberg JF, Coleman JS (2005). Discrimination training of phonemic contrasts enhances phonological processing in mainstream school children. Brain and Language.

[b14] Morrison S (1998). Computer applications: Earobics Pro. Child Language Teaching and Therapy.

[b27] MindWeavers (2002). Phonomena.

[b15] Pokorni JL, Worthington CK, Jamison PJ (2004). Phonological awareness intervention: Comparison of Fast ForWord, Earobics, and LiPS. Journal of Educational Research.

[b16] Rosen S (2003). Auditory processing in dyslexia and specific language impairment: Is there a deficit? What is its nature? Does it explain anything?. Journal of Phonetics.

[b17] Rosenberg JF, Moore DR (2003). Bulletin of the Royal College Speech Language Therapy.

[b25] Scientific Learning Corporation (1998). Fast ForWord Language.

[b18] Snowling MJ, Hulme C (2003). A critique of claims from Reynolds, Nicolson & Hambly (2003) that DDAT is an effective treatment for children with reading difficulties – ‘lies, damned lies and (inappropriate) statistics?’. Dyslexia.

[b19] Snowling MJ, Hulme C (2011). Evidence-based interventions for reading and language difficulties: Creating a virtuous circle. British Journal of Educational Psychology.

[b20] Tallal P (2004). Improving language and literacy is a matter of time. Nature Reviews Neuroscience.

[b21] Torgesen J, Wagner R, Rashotte C (1999). Test of word reading efficiency.

[b22] Troia GA (1999). Phonological awareness intervention research: A critical review of the experimental methodology. Reading Research Quarterly.

