# Supplementary material for: A Tale of Two Studies on Auditory Training in Children: A Response to the Claim that ‘Discrimination Training of Phonemic Contrasts Enhances Phonological Processing in Mainstream School Children’ by Moore, Rosenberg and Coleman (2005)
Source: Dyslexia. 2014 Jan 27;20(2):101–18. doi: 10.1002/dys.1470 (PMC4291104; doi:10.1002/dys.1470)
Supplement: Supplementary file 1 — Supporting info item [file dys0020-0101-sd1.docx]

**Table 1:** Comparison of post-training standard scores after controlling for pre-training standard scores on the Alliteration, Rhyme, and Spoonerisms subtests of the Phonological Assessment Battery (PhAB), for the Phoneme Discrimination (PD-Moore) and No-Intervention (NI-Moore) groups from Moore et al. (2005).

| Task | Group | Mean standard score  (S.D.) | Statistic | *p* value |
| --- | --- | --- | --- | --- |
| Alliteration | PD | 4.17 (7.00) |  |  |
|  | NI | 1.25 (6.00) | U (30) = 79.50 | .232 |
| Rhyme | PD | 111.00 (13.56) |  |  |
|  | NI | 86.50 (8.92) | *F*(1, 27) = 41.56 | < .001 |
| Spoonerisms | PD | 111.67 (12.03) |  |  |
|  | NI | 96.50 (12.04) | *F*(1, 27) = 29.04 | < .001 |

*Note* Data for the Alliteration task represent pre- to post-training standard score improvement. Data for the Rhyme and Spoonerisms tasks represent uncorrected post-training standard scores.

**Table 2:** Comparison of post-training standard scores after controlling for pre-training standard scores on the Alliteration, Rhyme, and Spoonerisms subtests of the PhAB for the Phoneme Discrimination (PD-Halliday) and No-Intervention (NI-Halliday) groups from Halliday et al. (2012).

| Task | Group | Mean standard score  (S.D.) | Statistic | *p* value |
| --- | --- | --- | --- | --- |
| Alliteration | PD | 2.18 (10.88) |  |  |
|  | NI | 2.68 (4.80) | U (44) = 263.50 | .596 |
| Rhyme | PD | 103.82 (15.26) |  |  |
|  | NI | 104.95 (15.03) | *F*(1, 41) = 0.77 | .384 |
| Spoonerisms | PD | 107.55 (14.92) |  |  |
|  | NI | 108.05 (12.76) | *F*(1, 41) = 0.00 | .990 |

*Note* Data for the Alliteration task represent pre- to post-training standard score improvement. Data for the Rhyme and Spoonerisms tasks represent uncorrected post-training standard scores.

**Table 3:** Comparison of post-training standard scores after controlling for pre-training standard scores on the Alliteration, Rhyme, and Spoonerisms subtests of the PhAB for the Phoneme Discrimination groups from Moore et al. (2005) (PD-Moore) and Halliday et al. (2012) (PD-Halliday).

| Task | | Study | Mean standard score  (S.D.) | | Statistic | *p* value |
| --- | --- | --- | --- | --- | --- | --- |
| Alliteration | | Moore | 4.17 (7.00) | |  |  |
|  | | Halliday | 2.18 (10.88) | | U (40) = 167.00 | .376 |
| Rhyme | | Moore | 111.00 (13.56) | |  |  |
|  | | Halliday | 103.82 (15.26) | | *F*(1, 37) = 7.63 | .009 |
| Spoonerisms | | Moore | 111.67 (12.03) | |  |  |
|  | | Halliday | 107.55 (14.92) | | *F*(1, 37) = 8.72 | .005 |
|  |  | | |  |  |  |

*Note* Data for the Alliteration task represent pre- to post-training standard score improvement. Data for the Rhyme and Spoonerisms tasks represent uncorrected post-training standard scores.

**Table 4:** Comparison of post-training standard scores after controlling for pre-training standard scores on the Alliteration, Rhyme, and Spoonerisms subtests of the PhAB for the No-Intervention groups from Moore et al. (2005) (NI-Moore) and Halliday et al. (2012) (NI-Halliday).

| Task | Study | Mean standard score  (S.D.) | Statistic | *p* value |
| --- | --- | --- | --- | --- |
| Alliteration | Moore | 1.25 (6.00) |  |  |
|  | Halliday | 2.68 (4.80) | U (34) = 161.50 | .292 |
| Rhyme | Moore | 86.50 (8.92) |  |  |
|  | Halliday | 104.95 (15.03) | *F*(1, 31) = 5.44 | .026 |
| Spoonerisms | Moore | 96.50 (12.04) |  |  |
|  | Halliday | 108.05 (12.76) | *F*(1, 31) = 14.64 | .001 |

*Note* Data for the Alliteration task represent pre- to post-training standard score improvement. Data for the Rhyme and Spoonerisms tasks represent uncorrected post-training standard scores.

**Table 5:** Comparison of post-training raw scores after controlling for pre-training raw scores (excluding non-native English speakers) on the Alliteration, Rhyme, and Spoonerisms subtests of the PhAB for the Phoneme Discrimination (PD-Moore) and No-Intervention (NI-Moore) groups from Moore et al. (2005).

| Task | Group | Mean raw score  (S.D.) | Statistic | *p* value |
| --- | --- | --- | --- | --- |
| Alliteration | PD | 0.71 (1.26) |  |  |
|  | NI | 0.33 (1.22) | U (26) = 62.50 | .458 |
| Rhyme | PD | 18.82 (2.27) |  |  |
|  | NI | 10.00 (3.16) | *F*(1, 23) = 85.07 | < .001 |
| Spoonerisms | PD | 20.41 (6.59) |  |  |
|  | NI | 12.33 (7.25) | *F*(1, 23) = 16.55 | < .001 |

*Note* Data for the Alliteration task represent pre- to post-training raw score improvement. Data for the Rhyme and Spoonerisms tasks represent uncorrected post-training raw scores.

**Table 6:** Comparison of post-training raw scores after controlling for pre-training raw scores (excluding non-native English speakers) on the Alliteration, Rhyme, and Spoonerisms subtests of the PhAB for the Phoneme Discrimination (PD-Halliday) and No-Intervention (NI-Halliday) groups from Halliday et al. (2012).

| Task | Group | Mean raw score  (S.D.) | Statistic | *p* value |
| --- | --- | --- | --- | --- |
| Alliteration | PD | 0.50 (3.07) |  |  |
|  | NI | 0.30 (0.73) | U (38) = 209.00 | .409 |
| Rhyme | PD | 17.22 (4.56) |  |  |
|  | NI | 17.75 (3.89) | *F*(1, 35) = 0.05 | .825 |
| Spoonerisms | PD | 19.89 (6.86) |  |  |
|  | NI | 20.00 (6.52) | *F*(1, 35) = 0.04 | .835 |

*Note* Data for the Alliteration task represent pre- to post-training raw score improvement. Data for the Rhyme and Spoonerisms tasks represent uncorrected post-training raw scores.

**Table 7:** Comparison of post-training raw scores after controlling for pre-training raw scores (excluding non-native English speakers) on the Alliteration, Rhyme, and Spoonerisms subtests of the PhAB for the Phoneme Discrimination groups from Moore et al. (2005) (PD-Moore) and Halliday et al. (2012) (PD-Halliday).

| Task | Study | Mean raw score  (S.D.) | Statistic | *p* value |
| --- | --- | --- | --- | --- |
| Alliteration | Moore | 0.71 (1.26) |  |  |
|  | Halliday | 0.50 (3.07) | U (35) = 115.50 | .219 |
| Rhyme | Moore | 18.82 (2.27) |  |  |
|  | Halliday | 17.22 (4.56) | *F*(1, 32) = 9.29 | .005 |
| Spoonerisms | Moore | 20.41 (6.59) |  |  |
|  | Halliday | 19.89 (6.86) | *F*(1, 32) = 5.61 | .024 |

*Note* Data for the Alliteration task represent pre- to post-training raw score improvement. Data for the Rhyme and Spoonerisms tasks represent uncorrected post-training raw scores.

**Table 8:** Comparison of post-training raw scores after controlling for pre-training raw scores (excluding non-native English speakers) on the Alliteration, Rhyme, and Spoonerisms subtests of the PhAB for the No-Intervention groups from Moore et al. (2005) (NI-Moore) and Halliday et al. (2012) (NI-Halliday).

| Task | Study | Mean raw score  (S.D.) | Statistic | *p* value |
| --- | --- | --- | --- | --- |
| Alliteration | Moore | 0.33 (1.22) |  |  |
|  | Halliday | 0.30 (0.73) | U (29) = 96.00 | .799 |
| Rhyme | Moore | 10.00 (3.16) |  |  |
|  | Halliday | 17.75 (3.89) | *F*(1, 26) = 17.82 | <.001 |
| Spoonerisms | Moore | 12.33 (7.25) |  |  |
|  | Halliday | 20.00 (6.52) | *F*(1, 26) = 11.89 | .002 |

*Note* Data for the Alliteration task represent pre- to post-training raw score improvement. Data for the Rhyme and Spoonerisms tasks represent uncorrected post-training raw scores.
